# Exogenous Application of Zinc to Mitigate the Salt Stress in *Vigna radiata* (L.) Wilczek—Evaluation of Physiological and Biochemical Processes

**DOI:** 10.3390/plants10051005

**Published:** 2021-05-18

**Authors:** Hassan S. Al-Zahrani, Hesham F. Alharby, Khalid Rehman Hakeem, Reiaz Ul Rehman

**Affiliations:** 1 Department of Biological Sciences, Faculty of Science, King Abdulaziz University, Jeddah 21589, Saudi Arabia; hsalzahrani@kau.edu.sa (H.S.A.-Z.); halharby@kau.edu.sa (H.F.A.); 2 Department of Bioresources, University of Kashmir, Hazratbal, Srinagar 190006, India; rreiazbiores@gmail.com

**Keywords:** antioxidants, phenylalanine ammonia-lyase, reactive oxygen species, salinity, tyrosine ammonia-lyase, zinc

## Abstract

Salt stress adversely affects the growth and productivity of crops. However, reports suggest that the application of various micronutrients could help the plant to cope with this stress. Hence, the objective of the study was to examine the effect of exogenous application of Zinc (Zn) on salt tolerance in *Vigna radiata* (L.) Wilczek (mungbean). Mungbean is considered to be an economically important crop and possess a strategic position in Southeast Asian countries for sustainable crop production. It is rich in quality proteins, minerals and vitamins. Three weeks old grown seedlings were subjected to NaCl (150 mM and 200 mM) alone or with Zn (250 µM). After 21 days of treatment, plants were harvested for investigating morphological, physiological and biochemical changes. We found that the Zn application mitigates the negative effect upon plant growth to a variable extent. This may be attributed to the increased shoot and root length, improved chlorophyll and carotenoid contents, enhanced total soluble sugar (TSS), total soluble protein (TSP) and proline accumulation, decreased H_2_O_2_ content and increased enzymatic antioxidant activities. Zn’s application improved the performance of the enzymes such as phenylalanine ammonia-lyase (PAL) and tyrosine ammonia-lyase (TAL) of the secondary metabolism, which resulted in the improvement of total phenol and flavonoids. The antioxidant activities such as 1,1diphenyl 2-picryl hydrazine (DPPH) and ferrous reducing antioxidant power assay (FRAP) of the plants also showed improved results in their salt only treatments. Furthermore, hydrogen peroxide (H_2_O_2_) and superoxide radical (SOD) scavenging activity were also improved upon the application of 250 µM zinc. Thus, Zn application in low doses offers promising potential for recovering plants suffering from salinity stress. In conclusion, we assume that zinc application improved salt tolerance in mungbean through the improvement of various physiological and photochemical processes which could prove to be useful in nutrient mediated management for crop improvement.

## 1. Introduction

Amongst abiotic stresses salt is one of the main cause for restraining the growth in crops [1]. The salinization of soil represents a major hurdle in agricultural expansion to increase crop production [2]. Increasing distribution of soil salinity causes great economic loss by a detrimental reduction in yield, thus posing a challenge for future food production. Nearly 71% of the earth’s area is salinized, 80% of which is due to natural processes and 20% is the result of anthropogenic activities [3]. The regions where soils are mainly affected by salt belong to arid and semi-arid, however, it is not restricted to these regions only as it has been recorded in a wide range of altitudes with different climatic conditions. The most prevalent soluble salt is NaCl followed by Na_2_SO_4_, CaSO_4_ and KCl [4].

Plants grown in the salt-affected soil undergo alterations in their physiology and metabolism at a cellular as well as whole-plant level. Based on their salt tolerance, plants are categorized into salt-tolerant, salt-sensitive and intermediately tolerant [5]. Salinity is deleterious to plant growth from the germination stage to the flowering and fruiting stage, resulting in decreased yield [6]. It causes osmotic and ionic stress by affecting its water relations [7]. Salinity stress causes the excessive generation of reactive oxygen species (ROS) and the balance between their generation and neutralization determines the plants survival [8]. This imbalance in ROS initiates the cell damage rapidly by acting as a trigger to a chain of reactions making the plant susceptible to the oxidative process leading to significant disruption and damage to cellular structures [9,10].

For protecting themselves plants counter these effects by their innate antioxidative defense machinery comprised mainly of enzymatic and non-enzymatic elements [11,12]. At times, plants’ self-defense is insufficient to combat the negatives of salt stress. To overcome these effects, some substances are exogenously applied to plants which include macronutrients, micronutrients and osmolytes [13,14]. Among the essential micronutrients, zinc (Zn) is reported to have a significant potential to alleviate plant abiotic stress [15]. Under unfavorable conditions, foliar application of Zn enhances plant growth and development [16], including mitigation of salt stress [17], accompanied by improvements in plant growth by boosting chlorophylls and consequently photosynthesis [18]. Zn protects the membranes against oxidative as well as peroxidative damages by stabilizing membrane integrity and permeability [19]. Zn acts as a vital component of various important enzymes, stabilizer of proteins including the DNA-binding proteins (Zn-fingers) [20,21]. It is vital to superoxide dismutase (SOD) as its Cu/Zn isoforms are most copious in plants which are considered the first line of defense amongst the antioxidative enzymes. The application of Zn decreases the activity of membrane-bound NADPH oxidase, reduces photo-oxidation, increases activities of superoxide dismutase, catalase and peroxidase [22,23]. Zn has a pivotal role in DNA replication and thus alters the expression of several genes [24].

Amongst the micronutrients the Zn deficiency is dire amongst the plants cultivated on saline soils and around the globe, more than 50% of soil used for agriculture has low levels of available zinc for plants [25]. Taking into account, the increasing population, decreasing agricultural land, food security and balanced diet, we chose to work with legume namely mungbean (*Vigna radiata* L.) which is significantly affected by salinity. Legumes are the second important human food crop after cereals and among legumes mungbean is an important ancient food crop in Asia, said to be domesticated particularly in India which is the largest producer of mungbean followed by Pakistan. Mungbean is short duration legume and farmers grow it in-between the main successive crops as it fixes the atmospheric nitrogen by forming symbiotic association with Rhizobium bacteria which is also beneficial for the succeeding crop. It also has efficient tolerance towards adverse climatic conditions but drought and salt stress reduces its productivity [26].

The present study focused on understanding plant behavior including morphological and physiological modulations in response to salinity and exogenously applied Zn. We aimed to assess the role of Zn in ameliorating the NaCl salt stress by improving various morphological and biochemical parameters in mungbean genotype—NCM-1 (NARC-Islamabad) classified as belonging to an arid region. The role of zinc in alleviating salt stress has been evaluated by measuring its effect on electrolyte leakage, lipid peroxidation and H_2_O_2_ content under NaCl stress. Effect of Zn on osmolytes and major antioxidant enzymes have been assessed in salt-stressed seedlings. The outcome of this study will help us in understanding the salt stress alleviation by Zn supplementation and its role in plant growth, development and protection across the world, especially in arid and semiarid regions.

## 2. Results

### 2.1. Effect of Salinity on Growth, Photosynthetic Pigments and Their Recovery by Exogenous Application of Zn

**Figure 1:**
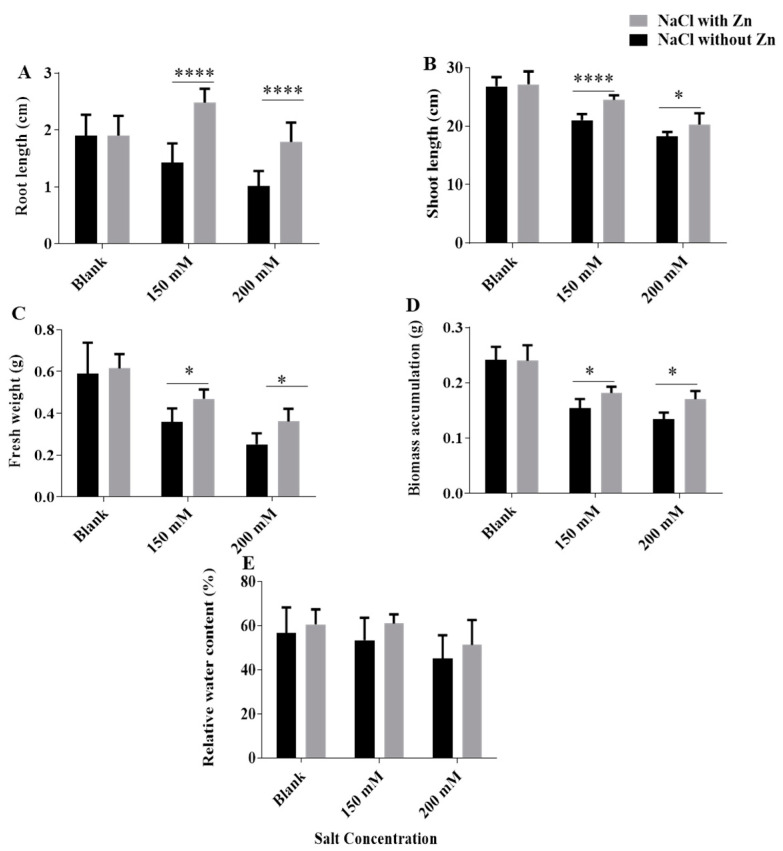
Effects of NaCl and Interactive effect of NaCl and Zn on (**A**) root length, (**B**) shoot length, (**C**) fresh weight, (**D**) biomass accumulation and (**E**) RWC of mungbean. The mean values with different letters across treatments are significantly different at *p* < 0.05. *, **** represent the significant difference between the two columns under the bar.

NaCl, 250 μM Zn and cumulative application of NaCl + Zn significantly affected the FW, DW, RWC, shoot and root length of seedlings (Figure 1). The fresh weight was decreased by 39.16% and 57.21% at 150 mM and 200 mM NaCl treatment, respectively, for salt-only trials. Addition of Zn exhibited an increase in fresh weight by 30.89% and 43.25% at 150 mM and 200 mM NaCl treatment, respectively. The dry weight was decreased by 32.98% and 44.50% at 150 mM and 200 mM NaCl treatment, respectively, for salt-only trials. Addition of Zn exhibited an increase in dry weight by 12.36% and 27.10% at 150 mM and 200 mM NaCl treatment, respectively. The relative water content (RWC) was reduced by 5.7% and 20.35% at 150 mM and 200 mM NaCl, respectively, for salt-only trials. Addition of Zn exhibited an increase in RWC by 14.30% and 13.78% at 150 mM and 200 mM NaCl treatment, respectively. The root length was reduced by 25.26% and 46.84% at 150 mM and 200 mM NaCl, respectively, for salt-only trials. Addition of Zn exhibited an increase in root length by 74.64% and 77.22% at 150 mM and 200 mM NaCl treatment, respectively. The shoot length was reduced by 21.56% and 31.71% at 150 mM and 200 mM NaCl, respectively, for salt-only trials. Addition of Zn exhibited an increase in shoot length by 16.52% and 10.90% at 150 mM and 200 mM NaCl treatment, respectively.

**Figure 2:**
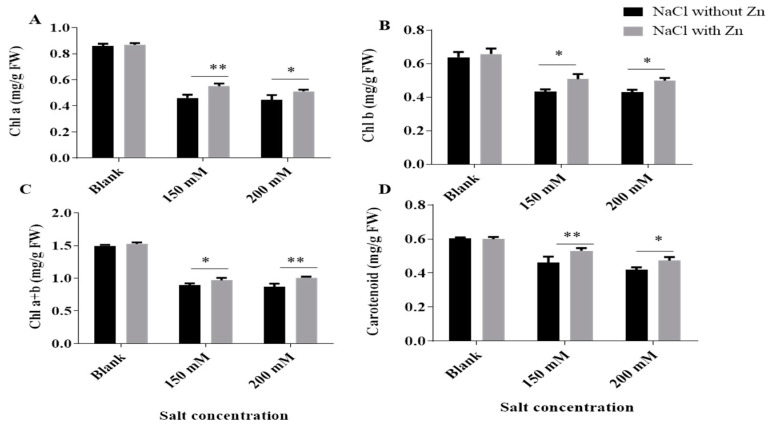
Effects of NaCl and interactive effect of NaCl and Zn on (**A**) chl a, (**B**) chl b, (**C**) chl a ± b and (**D**) carotenoids of mungbean. The mean values with different letters across treatments are significantly different at *p* < 005. *, ** represent the significant difference between the two columns under the bar.

The chl *a* (chlorophyll-a), chl *b* (chlorophyll-b) and carotenoid content of seedlings was reduced with salt treatments and an increase was observed by Zn supplementation (Figure 2). The total chlorophyll content was reduced by 40.33% and 41.65% at 150 mM and 200 mM NaCl, respectively, for salt-only trials. Addition of Zn exhibited an increase in total chlorophyll content by 8.40% and 15.68% at 150 mM and 200 mM NaCl treatment, respectively. The carotenoid content was reduced by 23.58% and 30.54% at 150 mM and 200 mM NaCl, respectively, for salt-only trials. Addition of Zn exhibited an increase in Carotenoid content by 14.99% and 12.96% at 150 mM and 200 mM NaCl treatment, respectively.

### 2.2. Effect of Salt Stress on Membrane Stability Index (MSI), Thiobarbituric Acid Reactive Species (TBARS) Content and Hydrogen Peroxide (H_2_O_2_) Content and Its Mitigation by Zn

**Figure 3:**
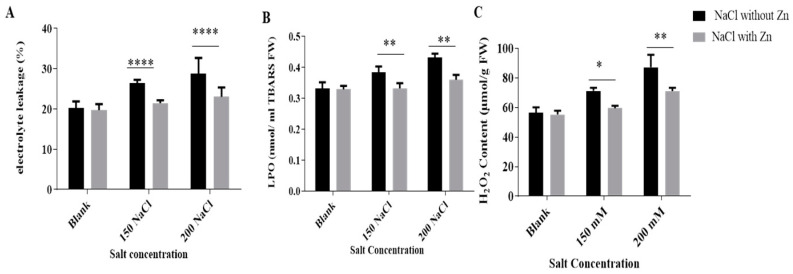
Effects of NaCl and interactive effect of NaCl and Zn on (**A**) MSI, (**B**) LPO and (**C**) H_2_O_2_ content of mungbean. The mean values with different letters across treatments are significantly different at *p* < 005. *, **, **** represent the significant difference between the two columns under the bar.

Membrane stability index (MSI), thiobarbituric acid reactive species (TBARS) content and hydrogen peroxide (H_2_O_2)_ content were significantly affected by NaCl, 250 μM Zn and cumulative application of NaCl and Zn (Figure 3). Membrane stability index (MSI)/electrolyte leakage was elevated with increasing salinity by 31.39% and 34.93% at 150 mM and 200 mM NaCl, respectively, for salt-only trials. The addition of Zn reduced electrolyte leakage by 20.89% and 18.15% at 150 mM and 200 mM NaCl treatment, respectively.

The salt stress results in lipid peroxidation (LPO) as reflected by the increased TBARS content. LPO increased salinity by 16.86% and 29.84% at 150 mM and 200 mM NaCl, respectively for salt-only trials. The addition of Zn reduced electrolyte leakage by 13.47% and 16.56% at 150 mM and 200 mM NaCl treatment, respectively. H_2_O_2_ content was increased salinity by 28.62% and 46.41% at 150 mM and 200 mM NaCl, respectively, for salt-only trials. The addition of Zn reduced H_2_O_2_ content by 16.45% and 18.28% at 150 mM and 200 mM NaCl treatment, respectively.

### 2.3. Effect of Salinity and Exogenously Applied Zn on Osmolytes (Proline, TSS and TSP)

**Figure 4:**
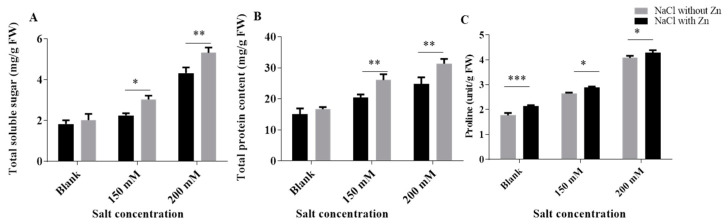
Effects of NaCl and interactive effect of NaCl and Zn on (**A**) TSS, (**B**) TPC and (**C**) proline content of mungbean. The mean values with different letters across treatments are significantly different at *p* < 0.05. *, **, *** represent the significant difference between the two columns under the bar.

NaCl, 250 μMZn and combined NaCl with Zn significantly affected the osmolytes content of mungbean seedlings (Figure 4). The proline content increased salinity by 23.56% and 40.85% at 150 mM and 200 mM NaCl, respectively, for salt-only trials. The addition of Zn increased the proline content by 9.05% and 23.03% at 150 mM and 200 mM NaCl treatment, respectively. The TSS content increased salinity by 11.48% and 42.26% at 150 mM and 200 mM NaCl, respectively, for salt-only trials. The addition of Zn increased the TSS content by 34.84% and 23.03% at 150 mM and 200 mM NaCl treatment, respectively. The TSP content increased salinity by 36% and 65.38% at 150 mM and 200 mM NaCl, respectively, for salt-only trials. The addition of Zn increased the TSS content by 27.56 and 26.04% at 150 mM and 200 mM NaCl treatment, respectively.

### 2.4. Effect of Salinity Stress and Zn on the Activity of Antioxidant Enzymes

**Figure 5:**
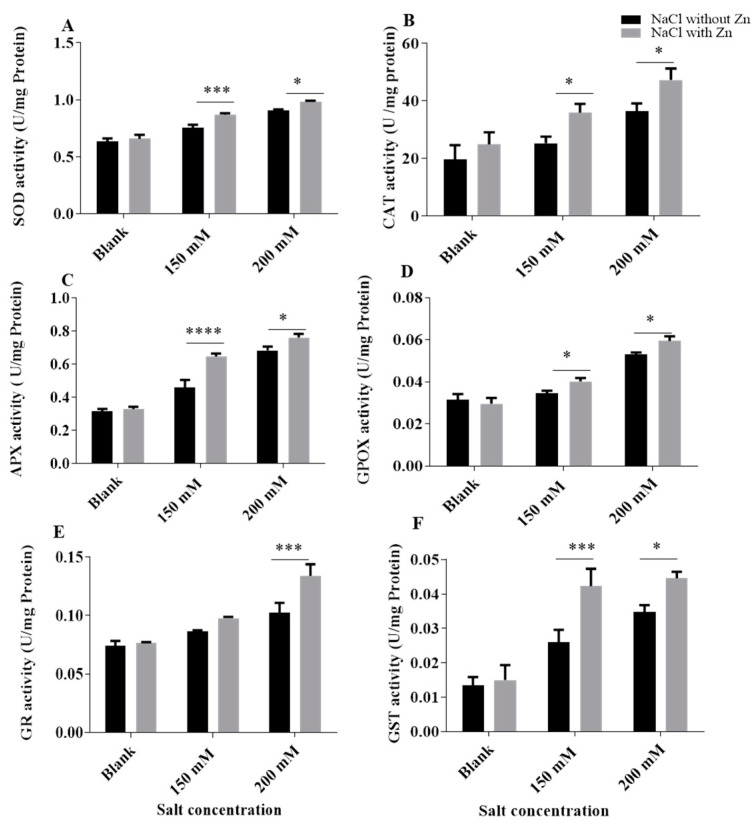
Effects of NaCl and interactive effect of NaCl and Zn on activity of (**A**) SOD, (**B**) CAT, (**C**) APX, (**D**) GPOX, (**E**) GR and (**F**) GST in mungbean. The mean values with different letters across treatments are significantly different at *p* < 0.05. *, ***, **** represent the significant difference between the two columns under the bar.

NaCl, 250 μM Zn and combined NaCl with Zn significantly affected the antioxidant enzymatic activity of mungbean seedlings (Figure 5). Superoxide dismutase (SOD) activity increased salinity by 18.77% and 43.24% at 150 mM and 200 mM NaCl, respectively, for salt-only trials. Addition of Zn increased the SOD activity by 15.45% and 8.52% at 150 mM and 200 mM NaCl treatment, respectively. Catalase (CAT) activity increased salinity by 28.83% and 85.39% at 150 mM and 200 mM NaCl, respectively, for salt-only trials. Addition of Zn increased the CAT activity by 42.08% and 29.84% at 150 mM and 200 mM NaCl treatment, respectively. Ascorbate peroxide (APX) activity increased salinity by 45.11% and 115.35% at 150 mM and 200 mM NaCl, respectively, for salt-only trials. Addition of Zn increased the APX activity by 40.79% and 11.57% at 150 mM and 200 mM NaCl treatment, respectively. Guaiacol peroxide (GPOX/POD) activity increased salinity by 9.19% and 68.28% at 150 mM and 200 mM NaCl, respectively, for salt-only trials. Addition of Zn increased the POD activity by 16.84% and 11.93% at 150 mM and 200 mM NaCl treatment, respectively. Glutathione-S-transferase (GST) activity increased salinity by 95% and 161% at 150 mM and 200 mM NaCl, respectively, for salt-only trials. Addition of Zn increased the GST activity by 62.82% and 28.16% at 150 mM and 200 mM NaCl treatment, respectively. Glutathione reductase (GR) activity increased salinity by 16.88% and 38.31% at 150 mM and 200 mM NaCl, respectively, for salt-only trials. Addition of Zn increased the GR activity by 12.86% and 30.85% at 150 mM and 200 mM NaCl treatment, respectively.

#### Multivariate Data Analysis

**Figure 6:**
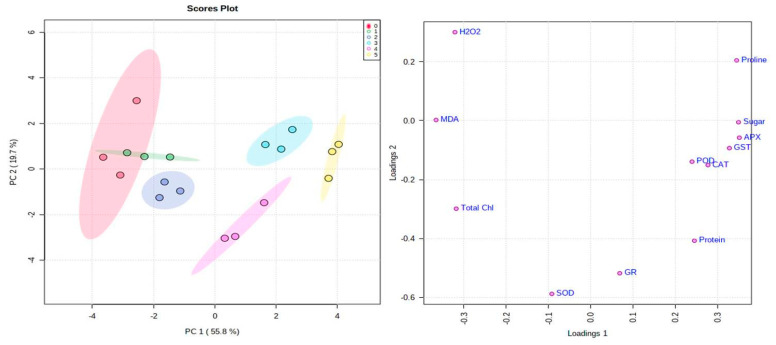
Effect of NaCl and Interactive effect of NaCl and Zn on principal component analysis (**A**) score plot and (**B**) loadings to understand the relationship among various metabolites in mungbean. The classes (0–5) in (**A**) refer to blank (0); Zn (1); 150 mM NaCl (2); 150 mM NaCl + Zn (3); 200 mM NaCl (4); 200 mM NaCl + Zn (5).

**Figure 7:**
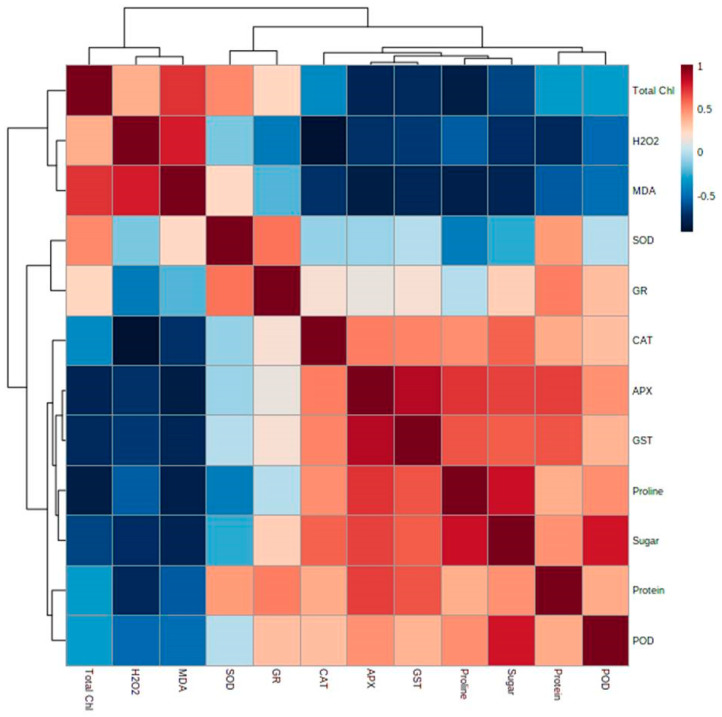
Effects of NaCl and interactive effect of NaCl and Zn on correlation analysis among the various physiological parameters of total chlorophyll (Total Chl). hydrogen peroxide (H_2_O_2_), lipid peroxidation (MDA), proline, protein, sugar, superoxide dismutase (SOD), catalase (CAT), ascorbate peroxidase (APX), guaiacol peroxidase (POD), glutathione reductase (GR) and glutathione-s-transferase (GST) in mungbean. Pearson’s correlation coefficient among data was analyzed using R scripts. The maroon color (1) indicates strong positive correlation among metabolites whereas blue (−0.5) indicates negative correlation among the metabolites.

Multivariate data analysis was conducted to measure the interrelationship among different metabolites, antioxidant enzymes in mungbean subjected to NaCl and NaCl + Zn treatments. To normalize the scale of abundance, the percent difference for every metabolite was log-transformed to base 2, preceding data analysis using MetaboAnalyst software 5.0 (https://www.metaboanalyst.ca, accessed on 1 May 2021). The unsupervised principal component analyses (PCA) including score plots clarify visualization of data and parallel comparison of the differentially modulated stress biomarkers in mungbeans (Figure 6A,B). Among various metabolites, proline, sugar and protein increased when subjected to 250 µM Zn + 200 mM NaCl indicating that Zn has an alleviating effect to mitigate the NaCl stress besides increased proline content suggests its crucial role in combating NaCl stress conditions. The principal component (PC) analysis depicted in Figure 6A,B showed that PC 1 and 2 explained 55.8% and 19.7% of the variability in the data. The angle between the vectors is an approximation of the correlation between the variables. A small angle indicates that the variables are positively correlated, an angle of 90 degrees indicates that the variables are not correlated, and an angle close to 180 degrees indicates that the variables are negatively correlated. The length of the line and its closeness to the circle indicate how well the plot represents the variable. It is, therefore, unwise to make inferences about relationships involving variables with poor representation. The covariance monoplot plots vectors pointing away from the origin to represent the original variables. The length of the line represents the variance of the variable, and the inner product between the vectors represents the covariance. A clear separation among various metabolites was found in the mungbean leaves subjected to 0 mM NaCl + 0 µM Zn; 250 µM Zn; 150 mM NaCl; 250 µM Zn + 150 mM NaCl; 200 mM NaCl; 250 µM Zn + 200 mM NaCl treatment in PC1 and PC2 according to the above measured parameters. The correlation analysis showed that the activities of SOD, APX, CAT, POD, GR, GST, proline, protein, sugar, MDA and total chlorophyll content had a positive correlation in mungbean leaves (Figure 7). Heat map analysis of various metabolites and antioxidant enzymes were conducted, and it was found that the antioxidant enzymes (CAT, POD, APX, GR and GST) and metabolites (proline, protein and sugar exhibited a significant increase (as marked in yellow) while as SOD, MDA and H_2_O_2_ (marked in red) showed a significant decrease when subjected to 250 µM Zn + 200 mM NaCl treatment (Supplementary Figure S2).

### 2.5. Effect of Salt and Salt with Zn on PAL (Phenylalanine Ammonia-Lyase) and TAL (Tyrosine Ammonia-Lyase)

**Figure 8:**
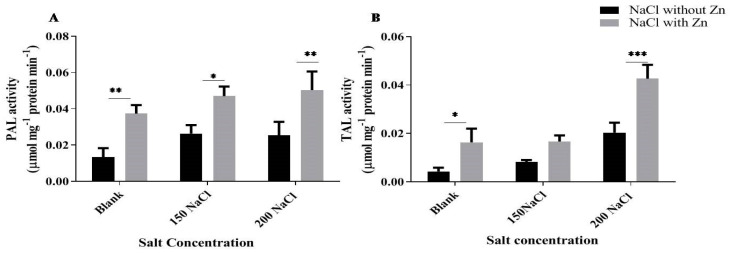
Effects of NaCl and interactive effect of NaCl and Zn on (**A**) phenylalanine ammonia-lyase and (**B**) tyrosine ammonia-lyase. Values represent mean ± SD, n = 3. The mean values with different letters across treatments are significantly different at *p* < 0.05; *, **, *** represent the significant difference between the two columns under the bar.

PAL activity was increased by 96.02% and 88.58% at 150 mM and 200 mM NaCl, respectively, for salt-only trials and application of Zn further enhanced it by 78.48% and 98.68% at 150 mM and 200 mM NaCl treatment, respectively. TAL activity was increased by 96.02% and 88.58% at 150 mM and 200 mM NaCl, respectively, for salt-only trials and application of Zn further enhanced it by 104.08% and 109.83% at 150 mM and 200 mM NaCl treatment, respectively (Figure 8).

### 2.6. Effect of Salt and Salt with Zn on TP (Total Phenol), TF (Total Flavonoid), TRP (Total Reducing Power), DPPH (2,2-Diphenyl-1-Picrylhydrazyl) and FRAP (Ferric Reducing Antioxidant Power Assay)

**Figure 9:**
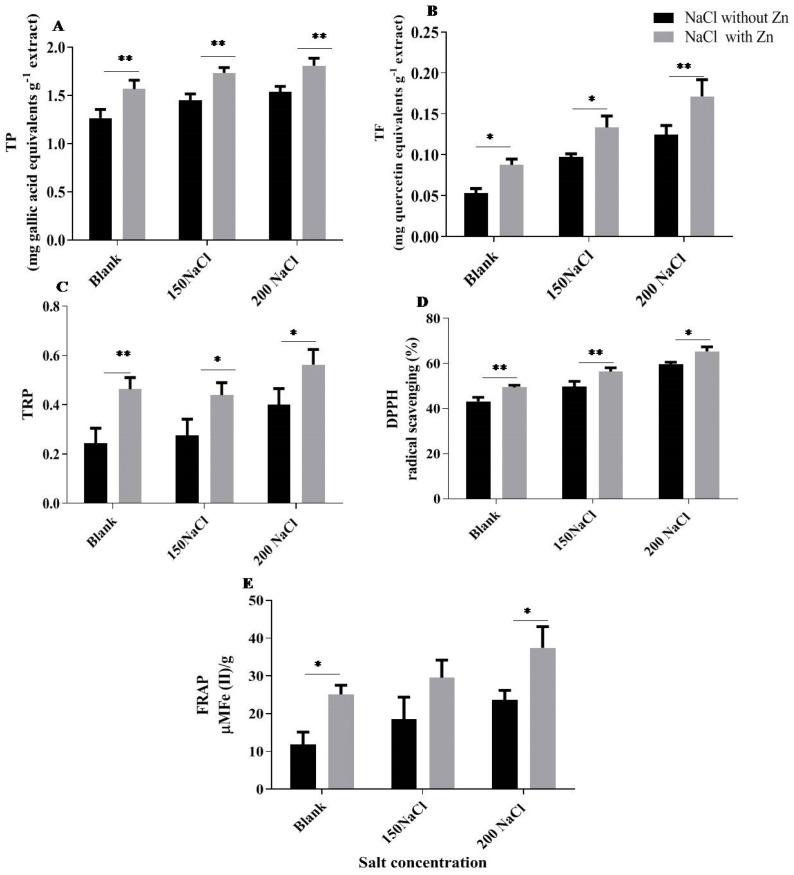
Effects of Zn; (**A**) total phenol, (**B**) total flavonoid content, (**C**) total reducing power, (**D**) DPPH and (**E**) FRAP antioxidant assays in mungbean. The mean values with different letters across treatments are significantly different at *p* < 0.05. *, ** represent the significant difference between the two columns under the bar.

The total phenol content of seedlings was increased by 14.71% and 21.57% at 150 mM and 200 mM NaCl, respectively, for salt-only trials. Application of Zn further enhanced it by 19.54% and 17.78% at 150 mM and 200 mM NaCl treatment, respectively (Figure 9). The total flavonoid content showed the same trend as total phenol; it increased by 83.14% and 133.70% at 150 mM and 200 mM NaCl, respectively, for salt-only trials. Application of Zn further increased by 36.84% and 37.80% at 150 mM and 200 mM NaCl treatment, respectively. The total reducing power was increased by 13.23% and 63.71% at 150 mM and 200 mM NaCl, respectively, for salt-only trials. Zn application further increased it by 109.52% and 8.51% at 150 mM and 200 mM NaCl treatment, respectively. DPPH was increased by 15.10% and 37.76% at 150 mM and 200 mM NaCl, respectively, compared to control, and Zn application further increased it by 13.49% and 9.75% at 150 mM and 200 mM NaCl treatment, respectively. FRAP was increased by 56.33% and 100.22% at 150 mM and 200 mM NaCl, respectively, compared to control, and Zn further increased it by 59.87% and 58.06% at 150 mM and 200 mM NaCl treatment, respectively (Figure 9).

## 3. Discussion

In plants, salt stress induces hyper-osmotic and hyper-ionic stress, ion toxicity (particularly caused by Cl^−^) in addition to dehydration [27]. Salinity poses deleterious effects on the photosynthesis process ranging from changes in pigment composition, restricted CO_2_ diffusion into the chloroplast, limitations on stomatal opening mediated by shoot-root-generated hormones and on the mesophyll transport of CO_2_, to alterations in leaf photochemistry and carbon metabolism [28]. Worldwide salinity is one of the major environmental limitations that significantly inhibit crop productivity, and the present study was executed to analyze the effect of Zn on the different mechanisms to combat NaCl toxicity in mungbean. Zn plays a key role in mediating different cellular functions [29]. The interaction of a micronutrient with a stressor is important for investigating, consideration and ultimately in refining plant defense strategies through various factors. In the current study, exogenously applied Zn showed a strong constructive association with key morphological and physiological components in mungbean under salinity. Noticeable changes were observed in fresh and dry weights as both declined due to salinity and Zn treatment alleviate the same (Figure 1). Our findings are in accordance with the previous study on salt-stressed *moringa* where a decrease in plant height, root length, branch number, fresh and dry weight of roots and shoots was observed [30,31].

Salinity stresses induce disturbances in chloroplast photochemistry. When the rate of absorption of light energy by photosynthetic pigments exceeds the rate of its consumption, the absorbed light energy accelerates the process of photoinhibition and decreased photosynthetic ability under salt stress [28]. Photosynthetic ability of plants also decreases due to the suppression of chlorophyll synthesis or decrease in the absorption of minerals needed for chlorophyll biosynthesis, which increased on zinc application and successively, increases plant height and yield, thereby playing a crucial role in biomass production.

In the current study, exogenous application of zinc showed pronounced increases in growth framework, i.e., length of root and shoot, fresh and dry weight. Similar, results were shown in cotton plants where zinc as nano-fertilizer showed increased growth in comparison to salt-treated plants [32]. In this study, salinity reduces the relative water content under salt stress (Figure 1E). It is believed that under salt stress, increased salt concentration hampers water uptake through the root [1]. According to Hafeez et al. [33], salinity induces loss of turgor due to decreased RWC, which obstruct the cell elongation process. Water deficiency might be collapsing all metabolic processes in the seedlings in saline conditions impeding their ability to grow in such conditions but zinc supplementation aids in survivability and helped in overcoming the effect of salinity. Our results are following Galal [34], who observed a decrease in RWC in *Hibiscus sabdariffa* L. suggesting it be amongst the important index of salt tolerance. Our results are also in accordance with Weisany et al. [29], suggesting the role of Zn in alleviating salt stress by positively regulating uptake as well as transport of water.

In the current study, salt stress results in a decrease of total chlorophyll in mungbean leaves (Figure 2), which is in accordance with the results of Alharby et al. [26], Weisany et al. [35], Hayat et al. [36], Iqbal et al. [37] and Ahmad et al. [38], in various plant species. Salinity obstructs the functioning of important enzymes responsible for the synthesis of photosynthetic pigments [39]. The modulation or destruction of these enzymes induces the chlorophyllase, responsible for chlorophyll degradation and ultimately reduced chlorophyll content [40]. While the Zn is believed to positively regulate magnesium transport, an important component of chlorophyll structure and hence results in increased chlorophyll content [35,41]. Increased chlorophyll contents are due to zinc which acts as a structural and catalytic component of proteins, enzymes and as a co-factor for the normal development of pigment biosynthesis [42].

Our results are in accordance with results in *Vigna radiate* [42] and in *B. juncea* [42] where chlorophyll increased with exogenous Zn application. The amelioration by Zn of declined chlorophyll which happened during salt stress might be due to zinc-induced shielding of the sulfhydryl group [35].

Salinity also induces a cascade of oxidative reaction resulting in the overproduction of ROS (Figure 3). Membranes are the main site of ROS attack which leads to severe lipid peroxidation, dimerization and polymerization of proteins, causing alteration of cell membranes [43].Under salinity, lipid peroxidation is a manifestation of damage and leakage of membranes [34,44]. In the current study, lipid peroxidation was evident under salt stress by increased ion leakage, TBARS content and H_2_O_2_ content. The increased ion leakage is associated with lipid peroxidation leading to membrane damage [45] and maintaining membrane integrity is considered a primary part of the salinity tolerance mechanism [46,47]. Our results are in accordance with the findings of Weisany et al. [29] and Rasool et al. [45]. The addition of zinc along with the salt mitigated deleterious effects of salt i.e., electrolyte leakage, TBARS content and H_2_O_2_ content declined significantly. It has been suggested that zinc alleviates membrane damage by maintaining its permeability to its proper range [48]. Zinc has a regulatory role on the Na^+^ and Cl^−^ uptake and translocation rate. So, in the salt-affected soils, zinc application could abate possible Na^+^ and Cl^−^ injuries such as ROS production and lipid peroxidation in the plant. The decrease in H_2_O_2_ was achieved upon Zn supplementation to seedlings growing with salt. Similar results were seen in salt-stressed eggplant where Zn lowered the H_2_O_2_ content [49]. Zinc-treated mungbean seedlings exhibited reduced TBARS and increased membrane stability, which validates the positive role of Zn in evading ROS-induced oxidative damage during salt stress. The results are parallel with the results of *Brassica juncea* under salt stress [38].

Osmolytes accumulates in response to salinity, wherein the accumulation of proline is a common metabolic process [50] (Figure 4). In the current study, increased proline accumulation was recorded on exposure to NaCl. These results are concomitant with the findings which reveal that extent of proline accumulation has a positive association/correlation with NaCl concentrations [51,52,53]. It is believed that salinity results in stomatal closure, which limits both the fixation of CO_2_ during photosynthesis as well as carbon reduction by the Calvin cycle. The decreased carbon reduction leads to the unavailability of NADP^+^ for electron acceptance so NADPH_2_ donates an electron to glutamate for proline biosynthesis and regenerates NADP^+^ for further electron acceptance [54,55]. The application of Zn further increased proline accumulation in salt-treated mungbean. Proline offers an important tolerance strategy by maintaining tissue water potential under environmental stresses including salinity [56,57,58]. Higher proline content in salt-stressed plants is maintained by increased activity of key enzymes involved in proline synthesis [59]. Proline protects the enzymatic function of different antioxidant enzymes and helps in free radical scavenging [60].

An enzymatic antioxidant system (SOD, POD, CAT, APX, GR AND GST) protects plants against oxidative damage of salinity [61,62,63] (Figure 5). Zinc also resulted in increased antioxidant enzyme activities. Increased SOD activity enables detoxification of more O^2-^ to H_2_O_2_, which is further detoxified to H_2_O. The conversion of H_2_O_2_ to H_2_O can be catalyzed by enzymes, such as CAT, APX and POX, which are also enhanced under Zn treatment. In the current study, the antioxidant enzyme activity significantly increases under salinity and this incline was even furthered with Zn treatment. The potential of tolerance against salinity in plants is determined by its antioxidant system [61]; as in our study, plants treated with Zn and salt had an efficient antioxidant system compared to salt-treated plants. Our results corroborate with the findings in maize [64] where Zn application stimulates antioxidant enzyme activities to protect plants from oxidative damage. Similarly, Ahmad et al. [38] found out zinc to be beneficial in alleviating salt stress in *Brassica Juncea*. SOD is the first line of defense opposing ROS and scavenges O_2−_. In the current study, the activity of SOD increased significantly during salt stress which is in accordance with the results in *Setaria italica* [45] and *Panicum milliaceum* [53]. Zn further amplified the SOD activity to alleviate the deleterious effect of salinity, which is in accordance with the findings in pistachio [65], sunflower [66] and eggplant [49] where Zn supplementation enhanced the SOD activity. Catalase scavenges H_2_O_2_ by converting H_2_O_2_ into H_2_O and 1/2 O_2_. In the current study, catalase activity increased in response to salinity and the addition of Zn further enhanced its activity, suggesting CAT elevation by Zn for improving H_2_O_2_ scavenging during stress. Our results are following Mallik et al. [67], where CAT activity increased in salt-tolerant plants under different salt treatments. They also suggest that under high salinity, various new isoforms of CAT are formed. Our finding with CAT is in accordance with the findings of Mittova et al. [68] in tomato. Zn supplementation further increased the CAT activity in salt-treated plants which is in accordance with the results of mustard [38]. Wani et al. [69] suggested that Zn is indirectly responsible for the increased activity of enzymes involved in eliminating H_2_O_2_. APX is another important enzyme that also has a pivotal role in H_2_O_2_ detoxification and concentration modulation in different cellular organelles [70]. In the current study, the activity of APX inclined in response to salt and Zn supplementation further stimulated the APX activity in the salt-treated mungbean plants. Our findings are in accordance with those obtained by Abbas [71], who suggested that increased APX activity might be due to increased H_2_O_2_ levels. Amiri et al. [65] also reported increased APX enzyme activity in Zn supplemented plants. Cakmak [72] suggested that Zn can facilitate the biosynthesis of antioxidant enzymes and alleviate the stress. Another important antioxidant enzyme is GST, which detoxifies xenobiotic compounds by mediating their binding to non-enzymatic antioxidants, such as tripeptide glutathione [10,73]. In this study, the activity of GST increases during salt stress and the Zn supplementation further enhanced the GST activity. Zn induced GST is responsible for rapid scavenging of radicals and has an important part in hormone homeostasis, stress responses and cell apoptosis [10,74] Our results showing increased GST activity are in agreement with the results of salt-stressed *Brassica juncea* [38] and in *Lycopersicon esculentum* [75].

POD catalyzes the reduction of H_2_O_2_ to water, and the presence of these enzymes has been established in several organisms including plants [76]. The POD activity in mungbean seedlings increased with increasing salinity as well as zinc supplementation. Our results are in coherence with *Triticum aestivum* [77]. It was exhibited in *Oryza sativa* that the expression of POD genes was obligatory for redox homeostasis [78,79]. Zn supplementation further enhanced the POD activity which is in agreement with the findings of salt-stressed *O. sativa* [80]. Another important antioxidant enzyme is GR, which has an essential role in protection against ROS through reduction of GSH [10,81,82]. Glutathione reductase (GR) is avital component of ascorbate-glutathione (AsA-GSH) cycle, which has a significant role in protection from ROS [83]. In the current study, the activity of GR increased during salt stress and Zn application significantly increased GR activity in wheat plants [84].

Further, the first principal component axis (PCA1) extracted 55.8% of variance for the total values of physiological change under NaCl stress in leaf samples of *Vigna radiata*. SOD, CAT, GR and protein had the highest contribution to the first axis. The second PC axis (PCA2) had lower significance (19.7% of variance) under salt stress and was mostly determined by the contributions of stress biomarkers. The length and direction of the vectors indicate the strength of the vector effect and correlation between vectors. Long vectors for all our parameters indicate that the vector greatly affected the results of the analysis. The cumulative percentage of PCA1 and PCA2 was 75.5% (Figure 6A,B). It was clear that the SOD, CAT, GR and protein grouped together with positive loading on the right lower side of the biplot, suggesting that these parameters are positive correlated with each other (Figure 7). Proline was observed on the right upper side of the biplot, while H_2_O_2_ and MDA were on the left upper portion of the biplot. The angles between different antioxidants are small, indicating that these parameters are positively correlated. On the biplot, sugar, APX segregate in opposite directions, indicating these factors are negatively correlated.

Salt stress leads to ROS’s overproduction [85] and requires the activation of a well-orchestrated antioxidant defense system to prevent ROS proliferation [86,87,88]. Phenolic compounds help in scavenging ROS under salinity stress [89,90]. The phenyl-propanoid biosynthetic pathway is central in forming different phenolic compounds [90,91]. Phenylalanine ammonium-lyase is a critical enzyme in the phenylpropanoid pathway and recognized as a marker of various abiotic stresses in different plant species [92]. It is an important enzyme that links primary and secondary (phenyl-propanoid) metabolism [92]. Salinity stress significantly affected PAL enzyme activity(Figure 8A), similar to results observed in chamomile herb under salt and heavy metal stress, where plants showed increased PAL activity after salt stress [93]. Exogenous application of Zn further improved the PAL activity to alleviate the deleterious effects of salinity. Our results are in correspondence with the finding of Luoet al. [94], where 2 mM Zn increased the PAL activity by 140% compared to control and are speculated to impart resistance to stress conditions [94]. PAL has been generally recognized as a marker of environmental stress in different plant species. Their increased activity is a typical response against biotic and abiotic stresses in plants, which stimulates secondary metabolite production. PAL and TAL are important enzymes of biosynthetic pathway of phenolic compounds. Phenolic compounds such as non-enzymatic antioxidants act as electron quenchers and are involved in suppression of ROS to alleviate the deleterious effects of salinity [93,95,96,97]. The present investigation revealed that the specific activity of TAL also increased with salt and Zn application (Figure 8B). Our results are in accordance with the findings of cluster bean where specific activity of TAL doubled after Zn treatment [98]. It is noteworthy to mention that Zn acts as a cofactor for PAL and TAL enzymes and therefore, these enzymes’ activities might have up-regulated after exogenous application of Zn.

Phenolic compounds play an essential role in scavenging excess ROS, notably phenolic acids and flavonoids assume a significant role. The present study observed that NaCl increased phenolic acids and flavonoids as a defense response in mungbean (Figure 9A,B) which corroborates with the study of Amaranth, where phenolic acids and flavonoids increased in response to salt stress [99]. However, in this study, we observed that upon applying exogenous Zn, the antioxidant activity increased as observed in pistachio [44]. Phenolic content increased after Zn application compared to salt treatment alone, with a maximum increase at 20 mg Zn kg^−1^ of soil [44]. Plants generally differ in their phenolic contents as genetic makeup and environment play an essential factor [100]. Phenolics enhance cell antioxidative capacity by inactivating free radicals and free radical generation by averting hydroperoxides’ decay [101,102]. Salt stress increases phenolic production to detoxify the ROS [103]. An improvement in the production of total phenol by exogenous application of Zn may be attributed to the development of nonstructural carbohydrate, enhancement in photosynthesis and proper translocation of photosynthate [104,105]. Flavonoids are low molecular weight polyphenols, which protect photosynthesizing cells and are involved in scavenging superoxide radicals, improving photosynthesis. In our study, the flavonoid content increased after salinity exposure. Similar results were found in Japanese catnip, where its content enhanced by low and moderate salinity levels but was constrained by higher levels [106]. Specific transcription factors associated with flavonol biosynthesis [107] are produced due to fluctuations in redox potential, and flavonol can serve as antioxidants in response to environmental stresses, including salinity stress [108,109]. Zn’s application (300 µM) further increased flavonoid content, but Zn’s role in enhancing the flavonoid content is still unclear. However, Zn acts as a chelator under salt stress [110], thus protecting the plant from ROS. Our results agree with the study on brown mustard, where it was found that Zn application increased phenol and flavonoid content [38]. The DPPH method is one of the effective methods for assessing the concentration of radical-scavenging. In mungbean, the increase in DPPH content was observed with an increase in salt concentration(Figure 9D), as observed in the study of sunflower callus extracts, where 300 mM NaCl concentration increased DPPH content by almost 1.12 times compared to control [111]. Zn addition further increased the DPPH content. DPPH activity under salt-treated is high because of phenolic compounds in higher amounts [112]. Another factor for increased activity of DPPH might be an increased amount of flavonoids [111,113,114]. Our results agree with the study on tobacco, where 0.24 mM µP-ZnO treatment showed significant inductions on the DPPH scavenging activity to control [115]. Moreover, salinity significantly enhanced the FRAP activity under salinity stress (Figure 9E), as reported in strawberries under salinity stress [116]. Our results showed a significant increase in FRAP with the application of Zn. Phenolic compounds have redox properties and act as hydrogen donators and singlet oxygen quenchers [117]. The redox potential of phenolic compounds helps in determining antioxidant potential. FRAP examines the reducing capability of an antioxidant with a ferric tripyridyltriazine complex and producing a shaded ferrous tripyridyltriazine.

## 4. Materials and Methods

### 4.1. Growth and Stress Conditions

This study is focused on the NCM-1 (NARC-Islamabad) genotype of mungbean (*Vigna radiata* (L.) Wilczek) and its seeds were procured from the Pakistan Agriculture Research, Islamabad. The seeds were soaked in 5% NaOCl solution for 10 min for sterilization. After sterilization, seeds were sown in plastic pots (22 cm diameter) containing 3 kg of moist acid-washed autoclaved sand. For nutrition, seedlings were supplemented with Hoagland’s nutrient medium of pH 6.5 [118]. These were grown under controlled conditions with 28 ± 1.5 °C at daytime and 22 ± 1.5 °C at nighttime, with a relative humidity of 60–70%. The treatments were started succeeding germination on the 21st day by making solutions in Hoagland’s medium to obtain the final concentration of 150 mM and 200 mM for NaCl and 250 μM for Zn, respectively (Supplementary Figure S1). Zn (250 μM) concentration was selected based on the tolerance index of Zn treatments ranging from 50 μM to 600 μM. Ten replicates were randomly used to determine growth parameters and three replicates were used for the biochemical and enzymatic analysis. Morphological parameters such as length, height, fresh and dry weight of roots and shoots were taken by measuring ten plants collected randomly. Weighing of material was performed individually for taking fresh weights (FW) followed by drying process for 72 h at 70 °C and finally, the dry weight (DW: biomass accumulation) was recorded by a precision balance. For calculating relative water content (RWC), the ten plants were weighed for fresh weight, and then these plants were placed in ddH_2_O for 24 h at 4 °C and weighed for turgid/swelled weight (SW) and finally, these plants were allowed to dry for 72 h at 70 °C and again weighed for the dry weight (DW). The values of FW, SW and DW from all treatments were used in the following equation.

RWC (%) = [(FW − DW)/(SW − DW)] × 100(1)

### 4.2. Estimation of Photosynthetic Pigments

The photosynthetic pigments chlorophylls (Chla:C₅₅H₇₂O₅N₄Mg, Chlb:C_55_H_70_MgN_4_O_6_) and carotenoids in fresh leaves were determined as described by the Lichtenthaler method [119]. The absorbance was recorded at 663 nm, 645 nm and 480 nm for chl a, chl b and carotenoids using Shimadzu UV-vis- spectrophotometer-1800 Japan and their quantities were indicated as mg g^−1^ FW.

### 4.3. Estimation of Membrane Stability Index (MSI)

The Tabot and Adams [120] method was followed for estimating electrolyte leakage percentage (ELP) or membrane stability index (MSI). Leaves sterilized 3 times with distilled water were cut into 1-cm^2^ sections and placed in stoppered vials with 10 mL distilled water. The vials were placed in a water bath at 40 °C for 30 min followed by recording the initial electrical conductivity of the solution (EC1). The vials were now subjected in a water bath to boiling temperature for 10min and then allowed to cool for recording the final electrical conductivity (EC2). The initial (EC1) and final (EC2) values were used for calculating ELP by the equation: [1 − (EC1/EC2)] × 100. The recordings were taken by using an electrical conductivity meter and ELP values indexed the changes in the permeability of cell membranes.

### 4.4. Determination of Lipid Peroxidation (LPO) Rate

Lipid peroxidation rate was assessed by evaluating the amount of total 2-thiobarbituric acid reactive substances (TBARS) and revealed as equivalents of malondialdehyde (MDA). TBARS was assessed by method of Cakmak and Horst [121]. The absorbance was taken at 532 nm and 600 nm and the MDA–TBA complex computed using extinction coefficient 155 mM^−1^ cm^−1^, and were, finally, expressed as nmol MDA g^−1^ FW.

### 4.5. Determination of Hydrogen Peroxide (H_2_O_2_) Content

Hydrogen peroxide (H_2_O_2_) content was assessed following the protocol of Velikova et al. [122] where frozen tissues (70 mg leaf) were coalesced in 5 mL of 0.1% (w/v) trichloroacetic acid (TCA) under ice-cold conditions. The coalesced mixture was centrifuged for 15 min at 12,000 rpm. To the 0.5 mL supernatant, 0.5 mL of 10 mM potassium phosphate buffer (pH 7) and 1 mL of 1 M KI were added, and readings were taken at 390 nm.

### 4.6. Estimation of Osmolytes

The osmolytes were assessed by methods: total soluble sugar (TSS) by Dey [123] free proline content by Bates et al. [124].The total soluble protein (TSP) was measured spectrophotometrically at 595 nm, using bovine serum albumin (BSA) standard following Bradford [125].

### 4.7. Enzymatic Assays

Fresh leaf tissue (200 mg) coalesced under chilled conditions with 3 mL extraction buffer (50 mM phosphate buffer pH 7.8; 1 mM EDTA-Na_2_; 1% PVP) in mortar and pestle. The coalesced mixture was centrifuged for half an hour at 13,000 rpm at 4 °C. The supernatant obtained was used for performing antioxidant enzyme assays.

#### 4.7.1. Determination of Superoxide Dismutase (SOD) Activity

The superoxide dismutase (SOD) activity was assessed according to its ability to inhibit photochemical reduction of nitro blue tetrazolium (NBT) [126] with slight modifications. The absorbance was measured at 560 nm against a blank using a UV-visible spectrophotometer and expressed as Umin^−1^ mg^−1^ protein.

#### 4.7.2. Determination of Catalase (CAT) Activity

Catalase (CAT) activity was assessed by the method described by Aebi [127]. This assay is based on decreased absorbance of H_2_O_2_ taken at 240 nm and activity computed using the extinction coefficient 0.036 mM^−1^cm^−1^ and expressed as Umin^−1^ mg^−1^ protein.

#### 4.7.3. Determination of Ascorbate Peroxidase (APX) Activity

Ascorbate peroxidase (APX) activity was assessed according to protocol by Nakano and Asada [128]. The enzyme activity was computed using extinction coefficient 2.8 mM^−1^ cm^−1^ and, later, expressed as Umin^−1^ mg^−1^ protein.

#### 4.7.4. Determination of Guaiacol Peroxidase (POD/GPOX) Activity

Guaiacol peroxidase (POD/GPOX) activity was assessed using guaiacol. Absorbance was recorded continually for the 90 s at 470 nm and enzyme activity was represented as an absorption unit per minute per mg protein (Umin^−1^ mg^−1^ protein) and activity was computed using the extinction coefficient of 26.6 mM^−1^cm^−1^.

#### 4.7.5. Determination of Glutathione-s-Transferase (GST) Activity

Glutathione-s-transferase (GST) activity was assessed following the protocol by Habig and Jacoby [129]. The absorbance was recorded at 340 nm and the activity computed using extinction coefficient 9.6 mM^−1^cm^−1^ and, finally, represented as unit per minute per mg protein (Umin^−1^ mg^−1^ protein).

#### 4.7.6. Determination of Glutathione-Reductase (GR) Activity

Estimation of glutathione reductase (GR) was performed by the method of Foyer and Halliwell [130] by following decreased absorbance at 340 nm due to NADPH oxidation (E = 6.2 mM^−1^ cm^−1^) and, finally, represented as unit per minute per mg protein (Umin^−1^ mg^−1^ protein).

### 4.8. Determination of Antioxidant Activity

Plants were collected from each treatment randomly and carefully washed with deionized water three times to remove sand from the surface of the roots; root length and shoot length was measured with the help of a ruler. Then the plant material was shade dried and then powdered and was stored in airtight bags until use. Next, 5 g of stored dried powder of leaf material was extracted with methanol using a shaker for 24 h at room temperature. The macerate was then filtered using Whatman’s filter paper. This supernatant was kept in airtight tubes and kept at 4 °C until further use.

#### 4.8.1. Determination of PAL and TAL Activity

The leaf material (0.5 g) was homogenized in 3 mL ice-cold sodium borate buffer (pH = 8.8) containing 1.4 mM 2-mercaptoethanol and 0.1 g of polyvinyl pyrrolidone. Then the mixture was centrifuged at 10,000 rpm for 25 min. To the aliquot (0.1 mL), add 0.1 M tri-sodium borate buffer (pH 8.5) and 0.5 mL of 12 mM L-phenylalanine. The final volume was then made 3 mL by adding deionized water and kept for incubation at 30 °C for 30 min. Then the increase in absorbance was recorded at 270 nm for 5 min. TAL activity was determined by measuring the amount of p-coumaric acid formed at 333 nm from L-tyrosine [131].

#### 4.8.2. Total Phenol and Total Flavonoid Content

Total phenol content was determined by the method given by Haard [131]. First, 0.1 mL of the prepared sample was taken and diluted with 0.4 mL of distilled water. Then freshly prepaid Folin–Ciocalteu Reagent (1 N) (0.5 mL) was added. After 3–4 min, 2 mL of sodium carbonate (20%) was added and thoroughly mixed. Then 2 mL ddH_2_O was added to each tube, making the final volume 5 mL. The tubes were kept in boiling water for 1 min. The absorbance was recorded at 650 nm using Shimadzu UV-1650 Spectrophotometer after cooling and kept in the dark for one hour. The color was read at 650 nm, using the method of Malik (Malik and Singh 1980). The sample concentration was calculated as gallic acid equivalent (GE), and phenolic content was expressed in mg/g of dry weight.

Total flavonoid content was determined by the colorimetric method developed by Woisky [132]. First, 0.1 mL of the already prepared sample was taken in test tubes and diluted by 0.4 mL of dd H_2_O, making the final volume 0.5 mL. Then methanolic aluminum chloride (AlCl_3_) was added, followed by 2 mL of potassium acetate (1M). Then ddH_2_O was added, making the final volume 5 mL. The mixture was incubated at room temperature for 30 min, and absorbance was recorded at 415 nm. Total flavonoid content was estimated using rutin as the standard. Flavanoid content was expressed as mg/g of dry extract.

#### 4.8.3. Determination of Total Reducing Power, DPPH and FRAP

The total reducing power was assayed by the method given by Yen [133]. An aliquot of 100 µL was mixed with 500 µL of 1% potassium ferricyanide. Then the reaction mixture was incubated at 50 °C for 20 min. Then 500 µl of 10% TAA were added and then centrifuged at 2500 rpm for 10 min. Then 1.5 mL of distilled water were added to the supernatant. Finally, 1% of ferric chloride (300 µL) was added, and absorbance was measured at 700 nm.

The antioxidative scavenging activity was determined using a stable free radical 1, 1 diphenyl 2-picryl hydrazine method. An aliquot (20–100 µL) of the extract was mixed with 1 mL of ethanol and 1 mL DPPH. The reaction mixture was incubated in the dark for 10 min, and the absorbance was read at 517 nm. The percentage inhibition activity was calculated by using the formula [134]:

Percentage inhibition = **A**_Control_ − **A**_Sample_/**A**_Control_ × 100

The FRAP assay was performed using Benzie’s [135] method with some modifications. An aliquot (10–50 µL) of extracts was mixed with 0.9 mL FRA reagent, and the mixture was kept in the dark for 10 min. The solution was diluted with ddH_2_O. Then, the absorbance of the blueish product, thus, formed was measured at 593 nm. Then the results were expressed in µM Fe(II)/g dry mass.

#### 4.8.4. Determination of Hydrogen Peroxide (H2O2) and Superoxide Radical (SOD) Scavenging Activity

Hydrogen peroxide scavenging activity was assayed by using the method of Ebrahimzadeh [136]. An aliquot of 10–50 µL of the extract was added to 600 µL of 40 mM H_2_O_2_ in phosphate buffer. Then phosphate buffer (pH 7.4) was added to make the final volume 5 mL. The reaction mixture was incubated at room temperature for 10 min, and absorbance was measured at 230 nm against the blank, where no H_2_O_2_ was added. The following formulae determined percentage inhibition:

Percentage inhibition = **A**_Control_ − **A**_Sample_/**A**_Control_ × 100

SOD scavenging activity was determined using the method given by Beauchamp [137]. First, 20–100 µL of extracts were mixed with nitro blue tetrazolium chloride (NBT) (0.1 mL). The reaction was initiated by 0.2 mL PMS (dissolved in 2.8 mL of 50 mM phosphate buffer having pH 7.6). The mixture was kept under fluorescent light for 20 min. After the formation of formazan color, the absorbance was measured at 562 nm. The percentage inhibition of SOD anion generation was estimated using the formula:

Percentage scavenging = (**A**_control_ − **A**_sample_)/**A**_control_ × 100

### 4.9. Statistical Analysis

The experiments were performed in triplicates (n  = 3) except for growth parameters FW, BA and RWC (where n  = 10). Data were treated with two-way ANOVA employing Graphpad Prism 6.0. The results were displayed as the arithmetic mean  ±  standard error (SE) and the statistical differences at 0.05 probability level were identified by Tukey’s post hoc test. The multivariate data analysis was performed by MetaboAnalyst software 5.0 (https://www.metaboanalyst.ca, accessed on 1 May 2021). To normalize the scale of abundance, the percent difference for every metabolite was log-transformed to base 2 preceding to data analysis using MetaboAnalyst software 5.0.

## 5. Conclusions

The present work illustrates the considerable distinction in cellular activity between the salt-treated and salt aided with zinc in mungbean. Under increased NaCl concentrations, salt-treated seedlings cultivar exhibit decreased root and shoot lengths, fresh weight, biomass accumulation and RWC in comparison to zinc treated salt-stressed plants. Salt treated plants also reveal high H_2_O_2_ content, which is associated with symptoms of oxidative damage, which go along with increased lipid peroxidation and electrolyte leakage. While the salt-stressed plants treated with Zn show less oxidative damage, comparatively. Likewise, the enzymatic antioxidant activity of Zn treated salt-stressed plants was much more than salt-stressed plants. Precisely, we can conclude that salinity and Zn combination elicited widespread physiological responses in mungbean plants, where Zn alleviates the harmful effects of salt and confer tolerance against the NaCl stress. The results obtained could be well thought-out as a nutrient administration tool for plants encountering stress conditions.

## Data Availability

Not applicable.

## Supplementary Materials

The following are available online at https://www.mdpi.com/article/10.3390/plants10051005/s1, Figure S1: Effects of NaCl and Interactive effect of NaCl and Zn on mungbean. Figure S2: Effects of NaCl and Interactive effect of NaCl and Zn on Heatmap based on the activities of total chlorophyll (total Chl), hydrogen peroxide (H2O2), lipid peroxidation (MDA), proline, protein, sugar, superoxide dismutase (SOD), catalase (CAT), ascorbate peroxidase (APX), peroxidase (POD), glutathione reductase (GR) and glutathione-s-transferase (GST) in mung bean. Heat map scale bars are shown (+1.5) to (−1.5) and the classes 0–5 represent the treatments in the study: Blank (0); Zn (1); 150 mM NaCl (2); 150 mM NaCl + Zn (3); 200 mM NaCl (4); 200 mM NaCl + Zn (5) The white color (1.5) indicates strong up-regulation of the metabolites, the red color (−1.5) indicates strong down-regulation of metabolites and the yellow colors (1) indicate moderate up-regulation of metabolites.
