# Supplementary material for: Exogenous Application of Zinc to Mitigate the Salt Stress in Vigna radiata (L.) Wilczek—Evaluation of Physiological and Biochemical Processes
Source: Plants (Basel). 2021 May 18;10(5):1005. doi: 10.3390/plants10051005 (PMC8157868; doi:10.3390/plants10051005)
Supplement: Supplementary file 1 [file plants-10-01005-s001.zip › plants-1199784-SI.pdf]

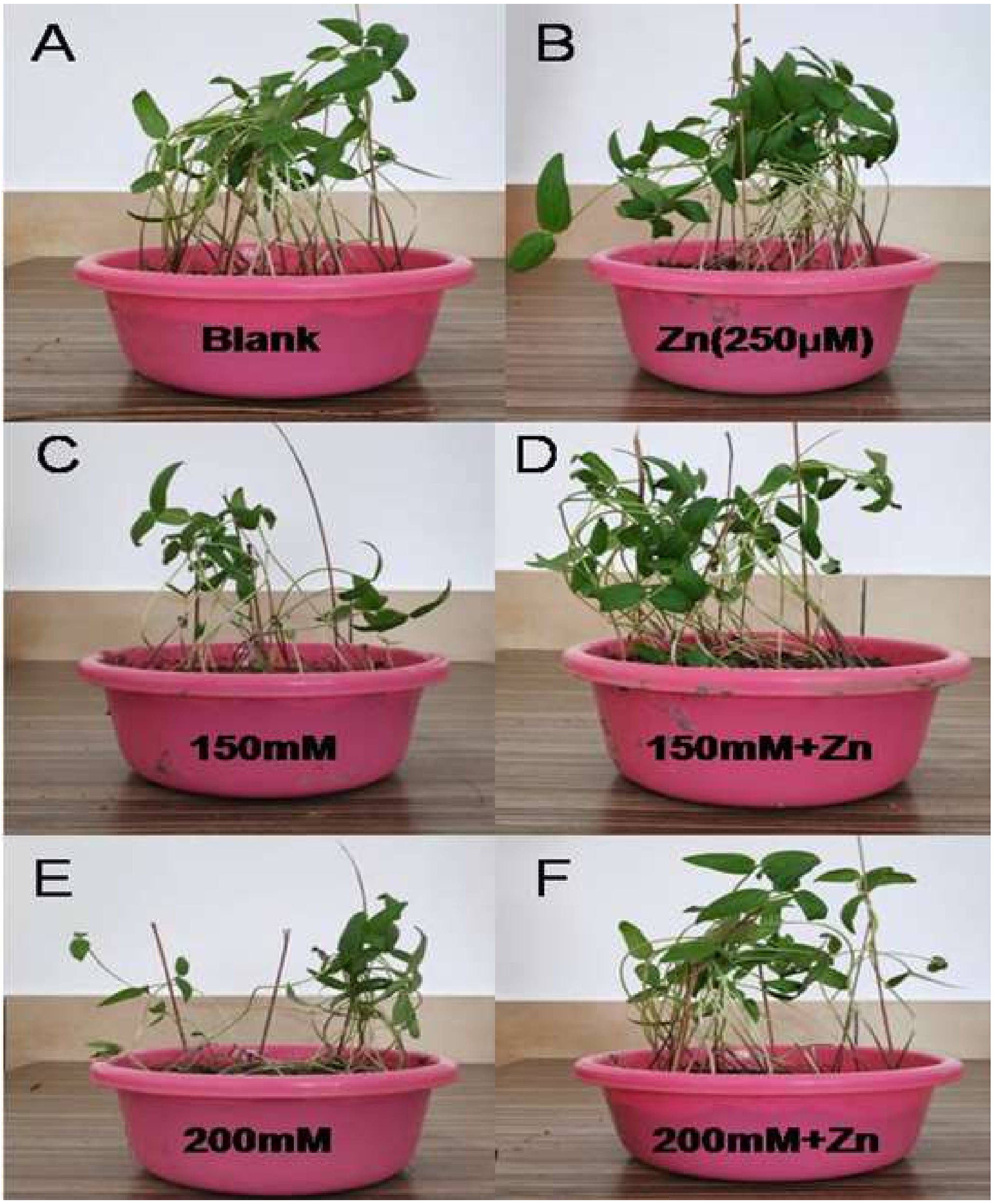

Figure S1. Effects of NaCl and Interactive effect of NaCl and Zn on Mungbean.

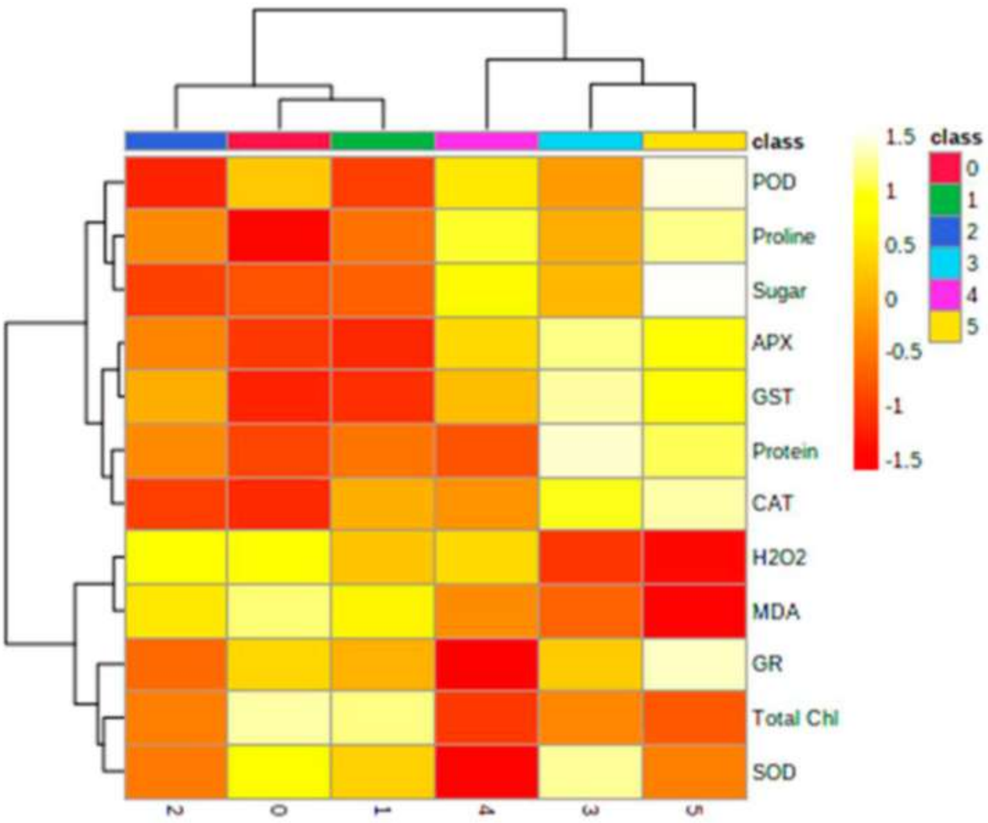

Figure S2. Effects of NaCl and Interactive effect of NaCl and Zn on Heatmap based on the activities of total chlorophyll (total Chl), hydrogen peroxide (H2O2), lipid peroxidation (MDA), proline, protein, sugar, superoxide dismutase (SOD), catalase (CAT), ascorbate peroxidase (APX), peroxidase (POD), glutathione reductase (GR), and glutathione-s-transferase (GST) in mung bean. Heat map scale bars are shown (+1.5) to (−1.5) and the classes 0–5 represent the treatments in the study. Blank (0); Zn (1); 150 mM NaCl (2); 150 mM NaCl + Zn (3); 200 nM NaCl (4); 200 mM NaCl + Zn (5). The white color (1.5) indicates strong up-regulation of the metabolites, the red color (-1.5) indicates strong down-regulation of metabolites and the yellow colors (1) indicate moderate up-regulation of metabolites.
